# Problem focused coping strategies and high self-compassion can be seen as protective factors to lower stress, negative emotional reactions to job and anxiety

**DOI:** 10.1192/j.eurpsy.2024.1182

**Published:** 2024-08-27

**Authors:** E. N. Gruber, S. Martic Biocina

**Affiliations:** ^1^Department R, Mental Health Centre Sct. Hans, Roskilde, Denmark; ^2^Department of Social Psychiatry, University Psychiatric Hospital Vrapče, Zagreb, Croatia

## Abstract

**Introduction:**

Previous research has shown that:

- problem focused coping strategies aim to change the reaction to the stressful situations
- self-compassion may reduce anxiety and depression. It is associated with happiness, it increases with age, it is negatively correlated with perceived stress and in older adults seems to be associated with higher levels of wisdom, integration, acceptance of one’s past life experiences and higher levels of meaning in life.
- job related low LPLA (Low pleasurable Low arousal emotions) and high LPHA (Low pleasurable High arousal emotions) levels corelate to depression, anxiety, and stress.

**Objectives:**

Case report of 60 years old computer scientist

**Methods:**

Psychiatric interview and scales:

Self-reported questionnaires: The Brief-COPE, The Self-Compassion Scale (SCS-SF), The Perceived Stress Scale (PSS 10), The Depression Anxiety Stress Scale (DASS-10), The Job-related Affective Well-being Scale (JAWS), The Subjective Happiness Scale (SHS), The Meaning in Life Questionnaire (MLQ)

**Results:**

60 years old male computer scientist, single, without children, multiple times a week in recreative activities and physical activities. He is not satisfied with close friendships, he sleeps 6 hours in day, he didn’t have traumatic experiences in life. He is not religious. He works 45 hours per week, from that 5-10 hours weekly works at the site of primary employment.

The Brief-COPE: High score for using problem focused coping strategies-acceptance, planning, positive reframing, and informational support. Maladaptive coping strategies used in lower grade: self-blame and self-distraction.

SCS-SF: Much higher levels of self-compassion than the norms established by previous research.

DASS-10: low.

SHS: lower happiness level than the norms established by previous research.

JAWS: High negative emotional reactions to job and low level of overall job-related affective wellbeing together with lower HPHA, higher LPHA, and lower LPLA in comparison to previous research. According to previous research this person is not satisfied with his job and have a lot of negative emotions regarding his job.

PSS 10: Levels of perceived stress are lower than the norms established by previous research.

MLQ: this person scored below 24 on the scale- presence of meaning and below 24 on the scale search for meaning. According to previous research person with this score do not feel their life has a valued meaning and purpose and they do not not actively explore that meaning or seeking meaning.

**Conclusions:**

Problem focused coping strategies and high self-compassion can be seen as protective factors to lower stress, negative emotional reactions to job and anxiety

**Disclosure of Interest:**

None Declared

